# Household Air Pollution and Respiratory Symptoms a Month Before and During the Stringent COVID-19 Lockdown Levels 5 and 4 in South Africa

**DOI:** 10.5334/aogh.3465

**Published:** 2022-01-10

**Authors:** Caradee Y. Wright, Thandi Kapwata, Nada Abdelatif, Chiara Batini, Bianca Wernecke, Zamantimande Kunene, Danielle A. Millar, Angela Mathee, Renée Street, Rikesh Panchal, Anna Hansell, Rebecca Cordell, Joshua Vande Hey

**Affiliations:** 1 Environment and Health Research Unit, South African Medical Research Council, 1 Soutpansberg Road, Pretoria, South Africa; 2 Department of Geography, Geoinformatics and Meteorology, University of Pretoria, Pretoria, South Africa; 3 Environment and Health Research Unit, South African Medical Research Council, 1 Soutpansberg Road, Johannesburg, South Africa; 4 Department of Environmental Health, Faculty of Health Sciences, University of Johannesburg, Johannesburg, South Africa; 5 Biostatistics Research Unit, South African Medical Research Council, 491 Peter Mokaba Ridge Road, Overport, Durban, South Africa; 6 Department of Health Sciences, University of Leicester, Leicester, United Kingdom; 7 Faculty of Health Sciences, University of the Witwatersrand, Johannesburg, South Africa; 8 Environment and Health Research Unit, South African Medical Research Council, 491 Peter Mokaba Ridge Road, Overport, Durban, South Africa; 9 Centre for Environmental Health and Sustainability, University of Leicester, University Road, Leicester, UK

## Abstract

**Background:**

Household air pollution (HAP) is associated with adverse human health impacts. During COVID-19 Lockdown Levels 5 and 4 (the most stringent levels), South Africans remained at home, potentially increasing their exposure to HAP.

**Objectives:**

To investigate changes in fuel use behaviours/patterns of use affecting HAP exposure and associated HAP-related respiratory health outcomes during COVID-19 Lockdown Levels 5 and 4.

**Methods:**

This was a cross-sectional online and telephonic survey of participants from an existing database. Logistic regression and McNemar’s test were used to analyse household-level data.

**Results:**

Among 2 505 participants, while electricity was the main energy source for cooking and heating the month before and during Lockdown Levels 5 and 4, some households used less electricity during Lockdown Levels 5 and 4 or switched to “dirty fuels.” One third of participants reported presence of environmental tobacco smoke in the home, a source of HAP associated with respiratory illnesses. Prevalence of HAP-related respiratory health outcomes were <10% (except dry cough). Majority of households reported cooking more, cleaning more and spending more time indoors during Lockdown Levels 5 and 4 – potentially exposed to HAP.

**Conclusion:**

Should South Africa return to Lockdown Levels 5 or 4, awareness raising about the risks associated with HAP as well as messaging information for prevention of exposure to HAP, including environmental tobacco smoke, and associated adverse health impacts will be necessary.

## Introduction

On March 11, 2020, the World Health Organization (WHO) declared the outbreak of severe acute respiratory syndrome coronavirus 2 (SARS-CoV-2), a novel coronavirus disease (COVID-19), a pandemic [1,2]. To slow the spread of COVID-19, international organisations, national and local governments, institutions, communities, and individuals adopted several public health and social measures [3,4]. In South Africa, this included the declaration of a National State of Emergency by the South African government [5] and implementation of a National Lockdown (March 26, 2020 to date) comprising a five-level COVID-19 alert system. The risk adjusted approach was guided by numerous criteria including level of infection and capacity of health care facilities.

On March 26, 2020, South Africa went into National Lockdown (Alert level 5) indicating “high COVID-19 spread with low health system readiness.” [6] The National Lockdown prevented free movement of people and prescribed social distancing, among other measures, to control and prevent the spread of the virus. Schools were closed and thus children, as well as employees working in non-essential services, spent majority of their time in a place of residence or dwelling.

In South Africa, about 14.5 million people rely on solid or “dirty” fuels (i.e., coal, wood, paraffin, etc.) as their main source of energy for cooking [7]. The type of fuel and how it is burned determines the emissions of the pollutants and their concentration in the air. The pollutant concentrations are influenced by, among other factors, the sources of the pollutants, as well as the ventilation inside a building. Personal exposure to air pollution is determined by how much a person inhales, at what concentration and for how long [8]. With the Lockdown Level 5 (the “strictest”) occurring during late summer/early autumn and as winter was approaching, domestic solid fuel burning was expected to increase for heating for thermal comfort and for cooking multiple meals daily for schoolchildren and employees who were housebound.

Few studies have considered household air pollution (HAP) risks in lockdown periods during the COVID-19 pandemic. A Spanish study found that mean daily HAP levels increased due to the lack of suitable ventilation and more intensive cleaning using harsh cleaning products and disinfectants [9]. In India, there was a concern that efforts to sustain clean cooking energy strategies would be derailed as people living in poverty may not be able to afford and access clean cooking fuels during lockdown [10]. Given the dearth of literature on this topic, and the concern for South Africans’ health in relation to HAP, here we present an investigation of fuel use behaviours/patterns of use affecting exposure to HAP and associated HAP-related respiratory health outcomes a month before and during Lockdown Levels 5 and 4. The three objectives of this study were: (1) to compare household fuel use patterns the month before and during Lockdown Levels 5 and 4; (2) to determine presence of adverse respiratory health symptoms among any household members in the home the month before and during Lockdown Levels 5 and 4; and (3) to consider whether fuel use patterns for heating and cooking and other HAP-related behaviours changed when comparing responses for the month before and during Lockdown Levels 5 and 4 (i.e., Level 5 from March 26, 2020 to May 1, 2020; adjusted Level 4 from May 1, 2020 to May 30, 2020).

## Methods

### Study approach

The study was designed as a retrospective study based on an online/telephonic survey questionnaire. The survey was conducted in four South African provinces, namely Gauteng, Western Cape, KwaZulu-Natal, and Eastern Cape. These provinces were selected for having the highest COVID-19 cases at the time of study preparation [11]. The study population included consenting adults residing in dwellings within the four provinces. Inclusion criteria included individuals aged 18 years or over and who were able to speak English, Zulu, Xhosa and/or Tswana.

Study participants were drawn from a market research company’s panel. Participants were passed through a double opt-in process that confers to the European Society for Opinion and Market Research (ESOMAR) guidelines [12]. This was followed by mobile authentication using a one-time password at registration. For the Computer-Assisted Telephone Interviews (CATI), participants from existing databases where the respondents were interviewed previously and opted to be recontacted in the future for research purposes were contacted to participate in the study. The sample was designed using Statistics South Africa and All Media Products Survey (AMPS) (i.e., re-weighted) population figures and quotas on demographics to match the South African national population demographics. Participants from a range of socio-economic-demographics were invited to participate. Online questionnaires were administered to those participants who could access online platforms and telephone surveys were done for those individuals who did not have access to online platforms (or mobile phone data, for example). Research ethics clearance for the study was granted by the South African Medical Research Ethics Committee (EC029–8/2020).

### Survey questionnaire

A short introduction (information sheet) was given at the start of the survey. After written or verbal consent was given for online survey or telephone interview, respectively, the survey/interview was initiated.

The questionnaire was designed on the lines of the questionnaire developed in the UK for national longitudinal cohort studies [13], bearing in mind South Africa’s unique socio-economic circumstances. Answers were captured electronically for the online survey and by the interviewer for the telephonic interview.

The questionnaire was divided into six parts (***Table 1***) namely: Part A: general participant information; Part B: socio-economic-demographic information; Part C: HAP indoors: the month before Lockdown Levels 5 and 4 (i.e., before March 26, 2020), and during Lockdown Levels 5 and 4 (i.e., from March 26, 2020 to May 30, 2020); Part D: respiratory symptoms/illnesses during Lockdown Levels 5 and 4; Part E: any presence of COVID-19 test/case in the household during Lockdown levels 5 and 4; and Part F: agency/choice and health promotion for informed decision-making and lifestyle changes. Finally, participants were thanked for completing the questionnaire and informed that there are health risks linked to being exposed to HAP, especially when cooking with coal, wood, or paraffin indoors. Moreover, if they had any concerns, or wanted more information, they were advised to contact their healthcare providers.

**Table 1:** Questionnaire main parts and questions.


PART	MAIN COMPONENTS

A	Preferred language for the survey, gender, ethnicity, and province of residence.

B	Type of dwelling in which the participant resides, if the dwelling had a ceiling and how many rooms in the dwelling, if there is running water and a flush toilet. Also, whether in the dwelling anyone in the household had a permanent job/income and/or received a social grant, how many people live in the dwelling (the month before Hard Lockdown), and how many of those children were under five years of age.

C	Main H the type of fuel used for heating and cooking, ventilation, presence of mould/dampness, animals, and environmental tobacco smoke and incense in the dwelling.

D	Anyone in the household had seen a nurse/doctor before (the month before) and during Hard Lockdown for the following health outcomes: dry cough, wet cough, hay fever, wheeze, shortness of breath.

E	Has anyone in the household been tested for COVID-19 Has anyone in the household been tested and found to be positive for COVID-19?

F	During Hard Lockdown, was it more difficult to make ends meet financially Are you aware of the health risks from burning wood/coal/paraffin indoors?


A Living Standards Measure (LSM) which is a means of segmenting South African people according to their living standards, where 1–4 equate to the lowest, 5–7 intermediate and 8–10 the highest LSM status was known from data in the market research company’s panel and also included in the dataset.

### Sample size calculation

To account for the different fuel use patterns across the four provinces (heating with solid fuels versus electricity) [14], it was hypothesized that there would be a change in domestic solid fuel burning post-Lockdown Levels 5 and 4 due to reduced incomes and more time spent at home cooking and heating, leading to higher electricity costs among those using electricity. It was assumed that there would be a 3% increase in solid fuel use, and a 0.1% increased use in electricity given the economic fallout from the pandemic, leading to a minimum total sample size of 1 148 participants, to achieve 80% power.

### Data management and statistical analyses

Data were converted from a .txt file to Stata version 16.1 [15]. Demographic characteristics of participants, fuel use patterns and respiratory symptoms the month before and during Lockdown Levels 5 and 4 were summarized using frequencies and percentages for categorical variables and means and standard deviations for continuous variables.

To meet objective 1, changes in fuel use patterns the month before and during Lockdown Levels 5 and 4 were examined using the generalized McNemar’s test, to account for both discordant as well concordant pairs i.e., participants who experienced changes in their fuel use as well as participants who have not changed their fuel use patterns. P-values < 0.05 were considered statistically significant.

To meet objective 2, respiratory symptoms were compared for the month before and during Lockdown Levels 5 and 4 using logistic regression.

Objective 3 (i.e., to consider whether fuel use patterns for heating and cooking and other HAP-related behaviours changed when comparing responses for the month before and during Lockdown Levels 5 and 4) was discussed in light of the findings from objectives 1 and 2 in the discussion. No analyses were done stratified by province since this was an overall analysis.

## Results

### Sample description

The survey was conducted between October 29, 2020 and December 2, 2020. ***Table 2*** provides the sample descriptive statistics by questionnaire variable. Majority of participants were Black African and female. About 40% of participants were from Gauteng likely since this is the most populous province in South Africa. There is also free Wi-Fi in certain public spaces of major cities, e.g., Tshwane Free Wi-Fi in Pretoria, possibly making it easier to complete the survey, compared for example to the Eastern Cape province where Internet access may have been a challenge. In this instance, CATI was applied to try and include more participants from areas without internet access.

**Table 2:** Study sample descriptive statistics (*N = 2 505*). Lockdown refers to Lockdown Levels 5 and 4.


VARIABLE	FREQUENCY N (%)

**Province of residence**	

Eastern Cape	284 (11)

Gauteng	1055 (42)

KwaZulu-Natal	662 (26)

Western Cape	504 (20)

**Gender**	

Female	1409 (56)

Male	1096 (44)

**Ethnicity**	

Black African	1804 (72)

Coloured (South African term for “of Mixed Ancestry”)	304 (12)

Indian/Asian	106 (4)

White	291 (12)

**Living Standard Measure (LSM)**	

LSM 1–4	250 (10)

LSM 5–7	599 (24)

LSM 8–10	1656 (66)

**Type of dwelling**	

House	1947 (78)

Flat	230 (9)

Shared accommodation, e.g., hostel	31 (1)

Shack	217 (9)

Room in backyard	58 (2)

Traditional hut	19 (1)

**Presence of ceiling in dwelling**	

Yes	1923 (77)

No	582 (23)

**Anyone in household have a permanent job before lockdown**	

Yes	1976 (79)

No	529 (21)

**Anyone in household receive any type of government social grant**	

Yes	1277 (51)

No	1228 (49)

**Running water inside dwelling**	

Yes	2075 (82)

No	430 (17)

**Flush toilet inside dwelling**	

Yes	2033 (81)

No	472 (19)

**Number of people live in dwelling before lockdown**	

0–4	1510 (60)

5–10	961 (38)

>10	34 (2)

**Were there 5-year-old and younger children who live in dwelling before lockdown**	

Yes	1098 (44)

No	1407 (56)


### Comparison of fuel use patterns and HAP exposure the month before and during Lockdown Levels 5 and 4

Overall, electricity was the most commonly used energy source for cooking and heating followed by gas (***Table 3***). Patterns of HAP exposure factors, excluding fuel use types for cooking and heating, were similar based on self-reported responses for questions pertaining to the month before and during Lockdown Levels 5 and 4. Most participants (about two-thirds) reported naturally ventilating their homes and did not have presence of mould, animals, incense burning or environmental tobacco smoke in their dwellings both before and during Lockdown Levels 5 and 4. However, about one in three participants did report presence of one or more of these HAP exposure factors. This being a particular concern for environmental tobacco smoke which is known to contribute to HAP and adverse respiratory health outcomes.

**Table 3:** Household air pollution sources including fuel use and related variables the month before and during Lockdown Levels 5 and 4 (*N = 2 505*).


VARIABLE	A MONTH BEFORE LOCKDOWN LEVELS 5 AND 4	DURING LOCKDOWN LEVELS 5 AND 4

	FREQUENCY	FREQUENCY

	N (%)	N (%)

**Main energy source for cooking**		

Coal	23 (1)	23 (1)

Electricity	1891 (75)	1857 (74)

Gas	385 (15)	410 (16)

Paraffin	109 (4)	117 (5)

Wood	97 (4)	98 (4)

**Main energy source for heating**		

Animal dung	12 (0.5)	9 (0.4)

Coal	45 (2)	58 (2)

Electricity	1887 (75)	1824 (73)

Gas	231 (9)	261 (10)

Paraffin	151 (6)	161 (6)

Wood	179 (7)	192 (8)

**Open windows and doors to ventilate**		

Yes	2313 (92)	2240 (89)

No	192 (8)	265 (11)

**Presence of mould/dampness in dwelling**		

Yes	669 (28)	673 (27)

No	1806 (72)	1832 (73)

**Presence of animals in dwelling**		

Yes	841 (34)	726 (29)

No	1664 (66)	1779 (71)

**Presence of ETS (smoke/vape) in dwelling**		

Yes	776 (31)	686 (27)

No	1729 (69)	1819 (73)

**Burn incense in dwelling**		

Yes	895 (36)	810 (32)

No	1610 (64)	1695 (68)


Changes in fuel use patterns for cooking for the month before versus during Lockdown Levels 5 and 4 were not statistically significantly different (***Table 4***). The main contributions were due to changes from electricity to gas (66 people, 2.6%) and gas to electricity (43 people, 1.7%).

**Table 4:** Generalized McNemar’s test for type of cooking fuel used before and during Lockdown Levels 5 and 4.


	COOKING FUEL DURING LOCKDOWN						

	*n (contribution to χ^2^)*						

		Coal	Electricity	Gas	Paraffin	Wood	Total

**Cooking fuel before lockdown**	**Coal**	16 (–)	1 (1.8)	3 (3)	2 (0.3)	1 (0.3)	23

	**Electricity**	4 (1.8)	1797 (–)	66 (4.9)	13 (1.8)	11 (0.2)	1891

	**Gas**	0 (3)	43 (4.9)	338 (–)	3 (0.2)	1 (0)	385

	**Paraffin**	1 (0.3)	7 (1.8)	2 (0.2)	95 (–)	4 (0)	109

	**Wood**	2 (0.3)	9 (0.2)	1 (0)	4 (0)	81 (–)	97

	**Total**	23	1857	410	117	98	2505

	***χ* ^2^**	**P-VALUE**					

Symmetry	12.52	0.252					

Stuart-Maxwell Marginal Homogeneity	8.18	0.085					


Changes in fuel use patterns for heating was significant (***Table 5***) though the actual numbers are small. The contributions were due to changes from coal to electricity (5 people, 0.2%), electricity to coal (14 people, 0.6%), electricity to gas (71 people, 2.8%), gas to electricity (44 people, 1.8%), electricity to paraffin (34 people, 1.4%) and paraffin to electricity (16 people, 0.6%).

**Table 5:** Generalized McNemar’s test for type of heating fuel used before and during Lockdown Levels 5 and 4.


	HEATING FUEL DURING LOCKDOWN							

	*n (contribution to χ^2^)*							

		Animal dung	Coal	Electricity	Gas	Paraffin	Wood	Total

**Heating fuel before lockdown**	**Animal dung**	5 (–)	1 (0.3)	1 (1)	1 (1)	1 (1)	3 (0.2)	12

	**Coal**	2 (0.3)	33 (–)	5 (4.3)	1 (0)	4 (0.8)	0 (2)	45

	**Electricity**	0 (1)	14 (4.3)	1735 (–)	71 (6.3)	34 (6.5)	33 (1.8)	1887

	**Gas**	0 (1)	1 (0)	44 (6.3)	184 (–)	1 (1)	1 (0)	231

	**Paraffin**	0 (1)	7 (0.8)	16 (6.5)	3 (1)	114 (–)	11 (0.9)	151

	**Wood**	2 (0.2)	2 (2)	23 (1.8)	1 (0)	7 (0.9)	144 (–)	179

	**Total**	9	58	1824	261	161	192	2505

	***χ^2^***	**P-VALUE**						

Symmetry	27.11	0.028						

Stuart-Maxwell Marginal Homogeneity	18.96	0.002						


### Self-reported presence of respiratory health outcomes the month before and during Lockdown Levels 5 and 4

Self-reported prevalence of doctor or nurse-diagnosed health outcomes was generally low at less than 10%, except for dry cough. There were statistically significant differences in the responses for dry cough, wet cough and hay fever for the month before versus during Lockdown Levels 5 and 4 (***Table 6***) when less of the health outcomes were experienced during the latter.

**Table 6:** Self-reported doctor or nurse-diagnosed respiratory health outcomes the month before and then during Lockdown Levels 5 and 4 (*N = 2 505*).


VARIABLE	A MONTH BEFORE LOCKDOWN LEVELS 5 AND 4	DURING LOCKDOWN LEVELS 5 AND 4	P–VALUE

	FREQUENCY	FREQUENCY	

	N (%)	N (%)	

**Doctor or nurse–diagnosed health outcomes**			

Dry cough	395 (16)	308 (12)	<0.001

Wet cough	218 (9)	154 (6)	<0.001

Hay fever	237 (9)	130 (5)	<0.001

Wheeze	72 (3)	59 (2)	0.128

Shortness of breath	112 (5)	106 (4)	0.599

**Anyone in household had a COVID test**			

Yes	–	888 (35)	–

No	–	1 617 (65)	–

**Anyone in household had a positive COVID**-**19 test result**			

Yes	–	188 (21)	–

No	–	700 (79)	–


One in three participants reported someone in the household having had a COVID-19 test during Lockdown levels 5 and 4. One in five respondents reported someone who had a positive COVID-19 test in the household during Lockdown levels 5 and 4.

### Changes in household air pollution-related behaviours and experiences during Lockdown Levels 5 and 4

Majority of households reported cooking more, cleaning more and spending more time indoors during Lockdown Levels 5 and 4 compared to the month before Lockdown Levels 5 and 4 (***Table 7***). More than 80% of participants reported finding it difficult to make financial ends meet.

**Table 7:** Lockdown Levels 5 and 4 experiences of questionnaire respondents: cooking, energy, crowding, cleaning, time indoors and financial difficulty (*N = 2 505*).


VARIABLE	FREQUENCY N (%)

**Cook more often during Lockdown**	

Yes	1 820 (73)

No	685 (27)

**Energy (e.g., electricity, wood, coal, etc.) become more expensive**	

Yes	1 838 (73)

No	667 (27)

**Fuel (e.g., electricity, wood, coal, etc.) become more difficult to get hold of**	

Yes	1 140 (46)

No	1 365 (54)

**More people staying in the dwelling during Lockdown**	

Yes	674 (27)

No	1 831 (73)

**Clean house more during Lockdown**	

More	1 779 (71)

Less	113 (5)

Same	613 (24)

**Spend more time indoors during Lockdown**	

Yes	2 336 (93)

No	169 (7)

**Difficult to make financial ends meet during Lockdown**	

No	437 (17)

Yes, somewhat	1 067 (43)

Yes, much more	1 001 (40)


## Discussion

The COVID-19 pandemic led to multiple, significant behavioural changes at individual and societal levels as countries devised various interventions and policies to slow the spread of the virus. In South Africa, a National Lockdown was implemented during which Lockdown Level 5 and 4 restrictions required all citizens, except essential workers, to stay at home.

Here, we aimed to compare household fuel use patterns the month before and during Lockdown Levels 5 and 4; to determine presence of HAP-related respiratory symptoms among any household members in the dwelling the month before and during Lockdown Levels 5 and 4; and consider whether fuel use patterns for heating and cooking and other behaviours changed (especially if they became riskier in terms of exposure to HAP) during Lockdown Levels 5 and 4.

From our study findings, electricity was the most commonly used energy source for household cooking and heating the month before and during Lockdown Levels 5 and 4. However, there was a slight (non-significant) change in electricity use for cooking with some (relatively few) households using electricity less during Lockdown Levels 5 and 4 compared to before Lockdown Level 5 (i.e., change from electricity to gas, or electricity to paraffin). This could be due to 83% of respondents reporting to struggle to make financial ends meet during Lockdown Levels 5 and 4, and possibly switching to alternate energy sources [16], as well as 73% of respondents reporting energy sources (i.e., electricity, wood, coal, etc.) becoming more expensive during this time, likely as a result of greater usage and not an increase in electricity price. By contrast, a study in Lagos, Nigeria, showed that there was an energy demand shift under a Lockdown Level 5 scenario, whereby 49% of the energy demand was from the residential sector – an increase on previous years demand. This was attributable to an increase in cooking (also reported in our study), and more people staying at and working from home [17].

While most participants (about two in three) reported naturally ventilating their homes and did not have presence of mould, animals, or incense burning (factors that can affect respiratory health among some individuals) in their dwellings both before and during Lockdown Levels 5 and 4, of particular concern was environmental tobacco smoke being present in one in three households both before and during Lockdown Levels 5 and 4. Environmental tobacco smoke is a contributor to HAP and is known to impact respiratory health especially among children, causing asthma, infection and wheeze [18,19].

In terms of objective 2, we found that self-reported prevalence of doctor or nurse-diagnosed respiratory health outcomes were less than 10% for both the month before and during Lockdown Levels 5 and 4. This could be as a result of recall bias, or be indicative of the Lockdown and health-seeking behaviours. A study in KwaZulu-Natal province showed no difference in daily clinic visitation at ambulatory clinics, suggesting that citizens continued to seek medical care during lockdown as done before Lockdown Level 5 [20]. However, other evidence showed that health-seeking behaviour declined in South Africa; compliance with child immunisation programmes declined [21] and reduced trauma cases were seen in some hospitals [22].

In terms of household behaviours, we saw majority of households reported cooking more, cleaning more and spending more time indoors during Lockdown Levels 5 and 4 compared to the month before. A study in rural Odisha, India, reported 41% of respondents having changed their household cleaning practices as a result of the COVID-19 outbreak or Lockdown [23]. Of these, 34% of respondents reported cleaning their house more frequently, and made mention of using water with phenyl, detergent, shampoo, or bleaching powder when washing floors. Similarly, 13% of respondents either started using or make use of more disinfectant or detergent when cleaning their house [23]. These findings are in keeping with the “at home lockdown vicious cycle” proposed by Domínguez-Amarillo et al [9].

With respect to objective 3, if we consider whether fuel use patterns for heating and cooking, and other behaviours related to HAP exposure changed during Lockdown Levels 5 and 4, we have insufficient evidence to confirm any significant changes, besides small changes in fuel type used for heating (with concern for the switch to “dirty” fuels like coal and paraffin) and more cooking and cleaning.

### Study limitations

We acknowledge that this was a survey-based questionnaire that relied on self-reporting hence there was a risk of recall (or response) bias. We tried to address this bias by requesting that participants report on doctor or nurse-related health symptoms and diseases and not self-reported symptoms/illnesses that may be more easily forgotten. We also tightened the time frame for occurrence of health symptoms and diseases to the month before and during Lockdown Levels 5 and 4 which were difficult periods in everyone’s lives and more likely recalled.

This study was conducted using non-probability quota sampling which does entail sampling bias. We set quotas for different types of respondents, mainly dependent on their home province, with more people surveyed from provinces affected by COVID-19 (at the time of project set-up) being surveyed. The aim of quota sampling was to control the composition of the final achieved sample at the design stage already. We did conduct the survey using probability sampling using a sample frame, and therefore there were no “non-responses” for which to account. A different sampling approach may be useful to apply in the future with a focus on ensuring good coverage of all LSM groups since we had few participants from the low LSM groups.

Self-reporting happened through the telephonic interviews as well as the online self-complete interviews. This is a limitation because of the current pandemic that requires contactless interviewing methods rather than face-to-face. The number of questions that one can have in an online or CATI survey is also restricted based on previous experience from the company who assisted with the data collection. This limited the questions we were able to ask and therefore prohibited some analyses, for example, comparing an individual who said they had COVID symptoms and whether or not they had a COVID test, followed by whether it was positive or negative. Future studies, preferably face-to-face, would permit for longer surveys where more detailed information would be gathered about all individuals separately living in the household.

Due to budget constraints and the cost of CATIs, the sample was skewed to the online survey which included a higher proportion of people from middle-to-high income groups. This affected the proportion of households using fuels associated with HAP, i.e., not electricity. Future studies should consider spending more funds on CATIs to gather data from lower-income households more likely to be exposed to high levels of HAP from the burning of solid or “dirty” fuels.

Our survey questionnaire did not ask about the urbanicity of the participant’s dwelling, i.e., urban, rural, peri-urban, peri-rural etc. and this should be included in future surveys. We also did not ask about specific type of geographical zone, such as township, suburb, inner city etc. and this information would be useful in future studies.

While several of the HAP respiratory health outcomes are similar to those for COVID-19, the questions about whether anyone in the household had had a COVID-19 test and any positive cases of COVID-19 in the household were included to consider presence or absence of COVID-19 in households. Future studies, with questions about specific HAP-related respiratory outcomes and specific COVID-19 health outcomes, as well as associated risk factors, might be useful if one wanted to assess the impact of HAP exposure on HAP-related respiratory outcomes and also to assess the impact of HAP exposure on COVID-19 specific symptoms/ill health effects.

## Conclusions

Should South Africa return to Lockdown Levels 5 and 4 (and perhaps even less stringent levels given the economic climate of the country) efforts will need to be made to raise awareness about the risks associated with HAP exposure, especially from the burning of “dirty” fuels for heating. Moreover, raising public awareness of the risks associated with environmental tobacco smoke is required. Future research should focus on groups most vulnerable to HAP exposure including people living in poverty, people living in townships, children, the elderly and people with pre-existing diseases.
